# High-Entropy Alloys: A Review of Emerging Sensing Materials for Next-Generation Flexible Electronics

**DOI:** 10.3390/ma19122655

**Published:** 2026-06-20

**Authors:** Huatan Chen, Zhongyi Yu, Yang Huang, Bofeng Li, Fangting Feng, Yuming Jiang, Yuting Duan, Gaofeng Zheng, Zungui Shao

**Affiliations:** 1 School of Mechanical and Automotive Engineering, Xiamen University of Technology, Xiamen 361024, China; chenht@xmut.edu.cn (H.C.); 18460089028@163.com (Z.Y.); 2522021023@stu.xmut.edu.cn (Y.H.); 2522021030@stu.xmut.edu.cn (B.L.); 18894625086@163.com (F.F.); 13799601321@163.com (Y.J.); 15159226091@163.com (Y.D.); 2 Pen-Tung Sah Institute of Micro-Nano Science and Technology, Xiamen University, Xiamen 361102, China; zheng_gf@xmu.edu.cn; 3 Fujian Provincial Key Laboratory of Terahertz Functional Devices and Intelligent Sensing, School of Mechanical Engineering and Automation, Fuzhou University, Fuzhou 350108, China

**Keywords:** high-entropy alloys, flexible sensor, mechanical properties, electrical properties, electrocatalytic properties

## Abstract

**Highlights:**

- HEAs provide a promising material platform for next-generation flexible electronics requiring high durability and reliability.
- Composition and microstructure engineering of HEAs open new pathways for high-performance, selective, and long-lasting sensors.
- Integration of HEAs into wearable and intelligent monitoring systems could significantly advance personalized healthcare and environmental safety.

**Abstract:**

High-entropy alloys (HEAs), composed of five or more principal elements in near-equimolar ratios, have emerged as a groundbreaking class of materials for next-generation flexible electronics. This review systematically examines the unique potential of HEAs as sensing materials, moving beyond their traditional role as structural components. We first elucidate the fundamental mechanisms—core effects including lattice distortion, sluggish diffusion, and the cocktail effect—that endow HEAs with an exceptional synergy of high strength, good ductility, tunable electrical resistivity, and superior electrocatalytic activity. Subsequently, we critically analyze the state-of-the-art strategies for processing HEA-based micro/nano structures, including mechanical alloying, wet-chemical synthesis, and non-equilibrium deposition techniques, with an emphasis on their compatibility with flexible substrates. The core of the review categorizes and discusses the latest advances in HEA-based flexible sensors for strain/stress, gas, and electrochemical (e.g., glucose, biomarkers, heavy metals) detection, highlighting the structure–property–performance relationships. Representative studies have demonstrated that HEA flexible strain sensors achieve a temperature coefficient of resistance as low as 45.59 ppm/K with no signal drift over 6000 stretching cycles; room-temperature hydrogen sensors reach a detection limit down to 31 ppb with a response time of 19 s; and non-enzymatic glucose sensors deliver a sensitivity up to 3043 μA·mM^−1^·cm^−2^. Finally, we summarize the key challenges—such as manufacturing scalability, long-term stability under dynamic deformation, and cost-effectiveness—and provide a forward-looking perspective on promising research directions, including high-throughput compositional screening, multi-functional sensor arrays, and the integration of machine learning for rational material design.

## 1. Introduction

With the rapid development of flexible electronics, wearable devices, and intelligent monitoring systems, flexible sensors have become core components for perceiving external physical, chemical, and biological signals. In practical application scenarios such as human motion detection, health monitoring, environmental perception, and industrial intelligent detection, flexible sensors are required to maintain stable signal output under complex service conditions, which puts forward higher comprehensive performance requirements for devices [1,2,3]. Currently, most flexible sensors still face certain challenges in practical applications. In terms of structural adaptability, it remains difficult to achieve highly accurate conformal contact with complex curved surfaces, and issues such as interfacial delamination or signal distortion may arise during deformation. Regarding mechanical stability, conventional conductive materials often exhibit limited resistance to repeated bending, stretching, and fatigue loading, making them prone to plastic deformation, fracture, or performance degradation after prolonged cyclic operation. As for sensing performance, there exists a notable trade-off between sensitivity and stability, and it is challenging to simultaneously achieve high sensitivity, a low detection limit, and fast response times [4,5,6]. More importantly, the existing mainstream flexible sensing materials, including bulk metals, binary/ternary alloys, conductive polymers, and liquid metals, cannot simultaneously meet the multi-dimensional demands of high sensitivity, mechanical robustness, long-term stability, and low-cost large-scale preparation, which restricts the further development and application of next-generation high-performance flexible sensors [7,8,9].

High-entropy alloys (HEAs), as an emerging class of metallic materials composed of multiple principal elements, depart from the conventional alloy design paradigm that typically relies on one or two dominant components. Owing to their distinctive structural features, HEAs are governed by four core effects—namely, the high-entropy effect, severe lattice distortion effect, sluggish diffusion effect, and cocktail effect—which collectively endow them with exceptional combinations of properties that are not readily achievable in traditional alloy systems [10,11,12]. The high entropy effect promotes the formation of simple and stable solid solution structures, avoiding the precipitation of brittle intermetallic compounds and ensuring structural stability; the severe lattice distortion effect induces abundant defects and unique electronic structures, which can significantly regulate electrical properties and sensing activity [13,14,15]; the sluggish diffusion effect endows HEAs with outstanding high-temperature stability, oxidation resistance, and corrosion resistance, adapting to harsh working environments; and the cocktail effect facilitates the synergistic optimization of multiple properties through the flexible adjustment of element types and ratios, so as to achieve the balance between mechanical flexibility, electrical conductivity, and sensing performance [16,17,18]. These unique advantages make HEAs perfectly match the performance requirements of flexible sensors for high sensitivity, mechanical robustness, and long-term stability, providing a new material solution to solve the current development dilemma of flexible sensors.

In recent years, the research on HEA-based flexible sensing materials and devices has developed rapidly, showing great application potential in strain/stress sensing, gas detection, electrochemical sensing, and other fields. However, there is still a lack of systematic summary and in-depth discussion on the structure–performance relationship, preparation process, and device integration of HEAs for flexible sensors. This review focuses on high-entropy alloys as emerging sensing materials for next-generation flexible electronics and intelligent monitoring, comprehensively reviews the regulation mechanisms of HEAs in mechanical, electromagnetic, and electrocatalytic properties, summarizes the controllable preparation of HEA micro/nano structures and the integration technology of flexible devices, and emphatically expounds the latest research progress of HEA-based flexible strain sensors, gas sensors, and electrochemical sensors. Finally, the performance advantages of HEAs are compared with those of traditional alloy materials, and the existing challenges and future development trends are prospected. This review aims to provide a comprehensive reference for the design, preparation, and application innovation of high-performance HEA-based flexible sensors, and promote the development and industrial application of next-generation flexible electronic devices.

## 2. High-Entropy Alloys as Sensing Materials

The superior performance of HEAs as sensing materials for microelectronic sensors primarily stems from the synergistic effects of their multi-principal-element configuration and the distinctive physicochemical characteristics induced by dimensional reduction [19,20]. To satisfy the rigorous operational demands of microelectronic sensors, chemical composition stands out as the fundamental factor governing the overall performance of HEA. Rational selection of constituent elements and modulation of atomic ratios effectively tailor lattice distortion, electronic structures and phase constituents, thereby tuning mechanical durability, electrical transport properties and electrocatalytic activity [21,22]. These inherent material characteristics further dictate core sensing parameters, including gauge factor, limit of detection, response speed and long-term stability, establishing an intrinsic composition–property–performance correlation for HEA-based sensing materials.

### 2.1. Tailoring the Mechanical Properties of HEAs

HEA sensing layers outperform conventional alloys in achieving a comprehensive balance of mechanical properties required for flexible electronics, maintaining a high strength limit while possessing outstanding flexibility [23,24,25]. Specifically, the multi-principal-element design in HEAs induces significant lattice distortion, which effectively impedes dislocation motion, resulting in strength and hardness far exceeding those of conventional pure metals and some alloys. This ensures that the sensitive layer does not easily undergo permanent deformation under significant strain. Furthermore, the high-entropy effect in some HEAs promotes the formation of simple solid solutions, preventing the precipitation of brittle intermetallic phases [26,27,28], while their multi-element solid-solution structure preserves excellent ductility for large deformation. Moreover, the unique microstructural features and sluggish diffusion effect of HEAs suppress defect accumulation and crack propagation under cyclic loading, delivering remarkably better fatigue resistance. Such superior mechanical synergy enables HEAs to serve as reliable functional layers for long-term working flexible sensors.

**Figure 1:**
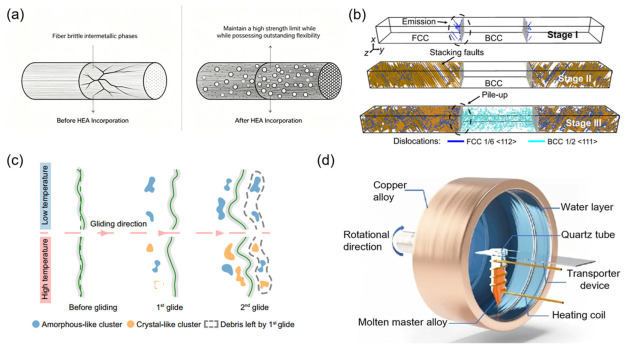
(**a**) Schematic Illustration of High-Entropy Alloy-Mediated Mechanical Property Modulation in Fibers. (**b**) Strain hardening curve of the as-printed EHEA and MD simulations showing the dislocation behaviour in the respective FCC and BCC lamellae at different stages of deformation [30]. (**c**) The schematic illustrationof the dynamic strengthening mechanism enabled by dislocation gliding induced local structural transition in a severely distorted crystal [31]. (**d**) Schematic diagram of the in-rotating-water spinning method [35].

Luo et al. [29] established an analytical model for TiTaHfZr refractory HEAs (RHEAs). Their study found that local lattice distortion, rather than chemical short-range order, is the key factor controlling local pinning points during the glide of edge dislocations, thereby determining the solid-solution strengthening effect. The model successfully predicted the yield strengths of various RHEAs, revealing that the macroscopic yield strength is governed by both the standard deviation of atomic radii and the number of constituent elements, while microscopically, it originates from dislocation pinning due to severe local distortion. Gao et al. [30] used the valence electron concentration as a design criterion to select the Al_19_Co_20_Fe_20_Ni_41_ eutectic HEA (EHEA). As shown in Figure 1b, they fabricated a specimen with a nanolayered hierarchical heterostructure via laser melting and employed molecular dynamics (MD) simulations with an embedded atom method (EAM) potential to study its deformation behavior. The material achieved a yield strength exceeding 1.3 GPa and a uniform elongation of 20%. The study revealed that the strength–ductility synergy originates from the multi-scale heterogeneous structure (including coherent nanoprecipitates, nanolayered structures, and hierarchical microstructural heterogeneities) and the activation of nanovoids within the hard BCC lamellae during deformation. These factors collectively promote sustained strain hardening and delayed fracture, providing a new pathway for designing high-performance structural materials. Wang et al. [31] prepared a novel single-phase B2 high-entropy intermetallic alloy, (CoNi)_50_(TiZrHf)_50_. As shown in Figure 1c, this alloy achieved a synergistic enhancement of strength and plasticity by leveraging a high density of lattice distortions. Unlike conventional intermetallic compounds, this alloy possesses a highly distorted lattice and complex chemical order, which activates dislocation slip and leads to high flow stress.

Furthermore, aligning with the trend towards flexibility, lightweight construction, and integration in flexible electronics, research into low-dimensional HEAs has garnered significant attention. Fibers, as a quintessential low-dimensional configuration, combine a high aspect ratio with excellent deformability, making them a key form for the flexible application of HEAs. Researchers have conducted systematic studies on the microstructural control of HEA fibers to optimize their mechanical properties. For instance, Huang’s group at Harbin Institute of Technology [32] used electric current treatment (ECT) on cold-drawn CoCrFeNi HEA microfibers by applying a specific density of direct current. This process successfully produced high-performance microfibers with both ultra-high strength and excellent plasticity. The ECT resulted in a uniform ultrafine-grained structure (~0.7 μm), an extremely low dislocation density, and a high density of the 9R phase. The treated microfibers exhibited a yield strength of 1.1 GPa and a uniform elongation of 43%. Such microfibers can effectively resist fracture and plastic deformation under repeated bending or tensile loading, making them ideal candidates for fabricating high-durability flexible sensors. Wang et al. [33] constructed an ultra-high density of nanotwins (with twin spacing as low as 1.2–2.5 nm) in CoCrFeMnNi HEA thin films via magnetron sputtering. Their study showed that when twin spacing exceeds 2 nm, nanotwins strengthen the material by hindering dislocation motion. Conversely, when twin spacing is below 2 nm, the deformation mechanism shifts to detwinning, which causes some softening but significantly enhances fatigue life. Li et al. [34] fabricated TaWTiVCr HEA thin films with varying elemental compositions via magnetron co-sputtering. As the Ti, V, and Cr content increased, the film structure gradually transitioned from a pure BCC phase to a mixture of BCC and amorphous phases. The films exhibited excellent mechanical properties due to the synergistic action of multiple mechanisms, including solid-solution strengthening, grain refinement, and encapsulation by an amorphous phase. Ma et al. [35] developed a soft magnetic HEA fiber (Fe_34_Co_29_Ni_29_Al_3_Ta_3_Si_2_) via a one-step rotating water bath spinning process, as shown in Figure 1d. By employing a strategy combining coarse grains (~48–57 μm) and coherent ordered L1_2_ nanoprecipitates (~18 nm in size, with a lattice mismatch of only −0.14%), they reduced domain wall pinning while improving dislocation mobility. This resulted in a tensile strength of 674 MPa and an elongation of 23%. During deformation, the as-spun fibers absorbed strain energy through dislocation proliferation and interactions, along with deformation-induced amorphization. In contrast, annealed fibers relied on deformation twins and the 9R phase to coordinate plasticity, thus overcoming the bottleneck of synergistically optimizing strength, plasticity, and magnetic properties in soft magnetic fibers.

In summary, through multi-principal-element design and microstructural engineering, HEAs achieve an excellent synergy between strength, plasticity, and fatigue resistance. Their unique lattice distortion effects and structural heterogeneities enable them to both resist deformation and maintain good toughness under complex mechanical loads, effectively overcoming the traditional trade-off between strength and ductility in metallic materials. This combination of high strength, good ductility, and fatigue resistance gives HEAs significant application potential in micro-sensors, as they can not only ensure the structural stability of the sensitive layer under repeated deformation but also guarantee long-term reliable electrical signal response.

### 2.2. Tailoring the Electrical Properties of HEAs

The emergence of HEA-based flexible electrodes not only fulfills the demands of high-performance devices due to their outstanding electrical sensitivity and wide detection range but also serves as a critical material for enhancing the reliability of flexible electronics, thanks to their excellent electrical conductivity. Leveraging the synergistic interactions among multiple elements, these electrodes effectively reduce signal transmission loss and ensure stable signal conduction, thereby significantly improving the operational reliability of flexible devices [36,37,38]. To better adapt to the service requirements of flexible electronics in complex application scenarios such as wearable devices, curved surface integration, and dynamic deformation, researchers have integrated high-performance alloy functional layers with flexible substrates, creating heterogeneous interfaces that enhance the devices’ electrical transport properties.

**Figure 2:**
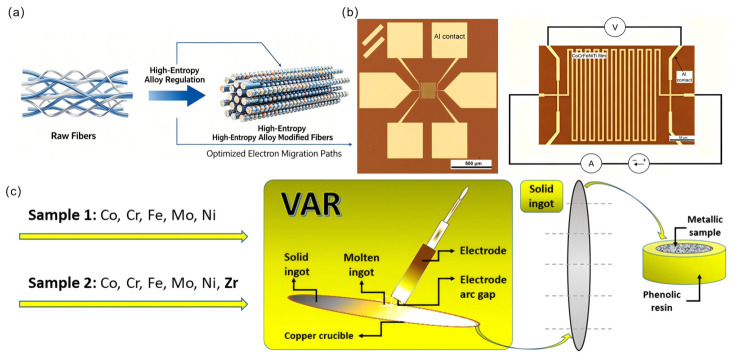
(**a**) HEA regulating the electrical conductivity of fibers. (**b**) Fabrication procedures of high-entropy CoCrFeNiTi-based thin-film resistor structure with Al electrodes [39]. (**c**) CoCrFeMoNi alloy sample obtention and preparation for the tests [43].

Poliakov et al. [39] fabricated amorphous/nanocrystalline CoCrFeNiTi_x_ HEA thin films via magnetron sputtering and patterned them into micro-resistors using photolithography, as shown in Figure 2b. The films, 130–230 nm thick, exhibited uniform elemental distribution and smooth surface morphology, with their amorphous structure remaining stable even after annealing at 400 °C. The room-temperature resistivity was 237 μΩ·cm, and the temperature coefficient of resistance (TCR) followed a linear trend from −78 ppm/°C to −6.6 ppm/°C in the −60 °C to 130 °C range, demonstrating low and stable temperature-dependent resistance. This study indicates the potential of CoCrFeNiTi_x_ HEA thin films as highly stable resistive components for microelectronic devices. Shen et al. [40] employed molecular dynamics (MD) simulations to investigate the modulation mechanism of nanogroove modification and defect engineering on the interfacial thermal resistance (ITR) of Si/Gr/HEA (FeNiCrCoCu) van der Waals heterostructures. Their work, combining MD simulations with phonon and interface structure analyses, systematically explored the influence of nanogrooves in the silicon substrate and defects in the graphene layer on ITR, revealing the underlying mechanisms from both phonon dynamics and interface structure perspectives. This provides a theoretical basis for interface engineering to address thermal resistance issues in HEA-based micro/nanoelectronic devices. Yang et al. [41] fabricated FeCrCoNiMn HEA nitride films on Si(100) substrates via magnetron sputtering, focusing on the effect of substrate temperature on the film’s electrical conductivity. Introducing nitrogen and adjusting the substrate temperature (40, 300, 500 °C) significantly improved conductivity. Nitrogen formed nanocrystalline metal nitrides with Cr and Mn, while Fe, Ni, and Co formed a Fe-Ni-Co nanocrystalline alloy phase. This resulted in a composite structure conducive to good electrical conductivity. With improved crystallinity and increased grain size, electron scattering at grain boundaries was reduced, leading to a significant increase in free electron density and mobility.

Although tuning the microstructure at heterogeneous interfaces represents an effective strategy for optimizing the electrical properties of HEAs, a more fundamental approach may lie in the direct manipulation of their composition. Through careful selection of the types and proportions of constituent principal elements, it is possible to proactively influence the phase constitution and electronic states of the alloy. This, in turn, allows for the precise tailoring of the material’s intrinsic electrical properties, potentially independent of the complexities associated with interface engineering. Le et al. [42] achieved multi-dimensional synergistic optimization of the electrical properties of a Ta-Nb-Hf-Zr-Ti HEA thin film by doping with carbon (C). Carbon atoms were interstitially incorporated into the BCC lattice without significantly altering the lattice constant, yet effectively enhancing the stability of the crystal structure. This doping approach not only increased the residual resistivity ratio and lowered the overall resistivity but also boosted the carrier density to 2.9 times that of the undoped film, demonstrating efficient control over electron scattering behavior. Furthermore, C doping did not alter the electron–phonon coupling constant but significantly enhanced the superconducting properties by increasing the critical temperature and upper critical field while reducing the magnetic penetration depth. The increased reflectivity in the low-energy region further optimized the optical conductivity, resulting in a comprehensive breakthrough in the film’s electrical performance across conventional, superconducting, and optical domains. Cabrera-Peña et al. [43] modified a CoCrFeMoNi HEA by doping with Zr. Their study showed that both the base and Zr-doped alloys exhibited a dendritic microstructure, but the addition of Zr led to grain refinement and a reduction in the alloy’s polarization resistance, as shown in Figure 2c. This result directly demonstrates the significant role of elemental doping in modulating properties related to electron transport. Li et al. [44] studied CoCrFeNiCu_x_ (x = 1, 2, 3, 4, 5) alloys and found that Cu content is a key factor determining the phase composition and microstructure. The electrical conductivity of the alloys was positively correlated with Cu content; higher Cu content led to lower resistivity, with CoCrFeNiCu_5_ exhibiting the best conductivity in the series. The overall difference in the coefficient of thermal expansion across the five alloys was small, indicating good dimensional stability over a wide temperature range and suitability for extreme environments with large temperature variations. The study ultimately identified CoCrFeNiCu_5_ HEA as possessing both excellent comprehensive mechanical properties and high electrical conductivity.

From the perspective of charge transport behavior, the unique structural features of HEA play a decisive role. Severe lattice distortion creates disordered atomic arrangements and strong potential fluctuations, which significantly enhance electron scattering and tailor material resistivity. The synergistic effect of multiple elements modulates electronic states and carrier density, enabling flexible regulation of electrical properties. In addition, grain size, defects, and phase interfaces are critical factors affecting carrier migration. Rational microstructure control can reduce invalid electron scattering and form stable charge transport channels, which is essential for HEAs to maintain a reliable electrical response under long-term deformation.

The enhancement of the electrical conductivity of HEA is not the result of a single mechanism, but rather the synergistic outcome of multiple factors. Element types and content ratios significantly govern the mechanical and electrical properties of HEAs. Transition metal elements such as Co, Cr, Fe and Ni can induce severe lattice distortion, which enhances strength and fatigue resistance but simultaneously increases material resistivity. Appropriately increasing the content of ductile elements can improve stretchability, yet excessive addition will reduce structural stability under cyclic deformation.

For electrical performance, introducing high-conductivity elements like Cu effectively lowers resistivity, while refractory metal elements such as Ta, W and Nb help reduce TCR. Tuning elemental ratios can balance resistivity and TCR, so as to obtain HEAs suitable for high-precision strain sensing.

### 2.3. Tailoring the Electrocatalytic Properties of HEAs

Target-detection electrochemical sensors serve as key devices in fields such as biomedicine, environmental monitoring, food safety, and industrial process analysis. The upper limit of their sensing performance is collectively influenced by four primary factors: electrocatalytic activity, interfacial charge transfer efficiency, selectivity, and long-term stability [45,46,47]. Within this context, the sensitive electrode material plays a central role in signal recognition and transduction. HEAs have shown promise in electrocatalytic sensing applications, owing to their unique physicochemical characteristics. From a compositional perspective, their nearly limitless tunability enables the on-demand optimization of adsorption energies at active sites. Structurally, the presence of substantial lattice distortion and abundant defect features can effectively modulate the electronic structure and reduce surface reaction energy barriers. At the interfacial level, their favorable conductivity, structural robustness, and resistance to interference collectively contribute to an efficient, selective, and durable catalytic response toward target molecules [48,49,50].

As the fundamental factor determining electrocatalytic behaviors, elemental composition plays a dominant role in regulating the overall sensing performance of HEAs. The elemental composition of HEAs dictates their electrocatalytic performance for gas and electrochemical sensing. Precious metal elements (Pt, Pd, Ru) provide abundant active sites and optimize the adsorption energy of target molecules, improving sensitivity and response speed. Non-noble transition metals (Fe, Co, Ni, Mn) synergistically adjust the d-band center, reduce reaction energy barriers and cut material costs. Minor doping of elements like Mo and Zr can further modulate surface electronic states, enhance anti-poisoning ability and selectivity. Different component combinations tailor the catalytic characteristics, thus determining the final sensing performance.

Beyond direct sensing applications, a large number of reported HEA electrocatalysts were originally designed for energy-related reactions, and these studies are also highly instructive for sensor development. It is worth noting that many HEA electrocatalysts are initially developed for the hydrogen evolution reaction (HER), oxygen evolution reaction (OER) and oxygen reduction reaction (ORR) in energy conversion systems. These studies are highly relevant to electrochemical sensing. Fundamentally, both electrocatalytic energy reactions and sensing reactions rely on the same core characteristics: abundant active sites, optimized intermediate adsorption energy, fast interfacial charge transfer and long-term structural stability. The strategies for tuning composition, microstructure, defects and electronic structures that have proven effective in energy electrocatalysis can be directly transferred to design high-performance HEA sensing electrodes. In addition, the excellent anti-corrosion and anti-poisoning capability of HEAs verified in harsh electrolytic environments also guarantees reliable operation of sensors in complex practical samples.

**Figure 3:**
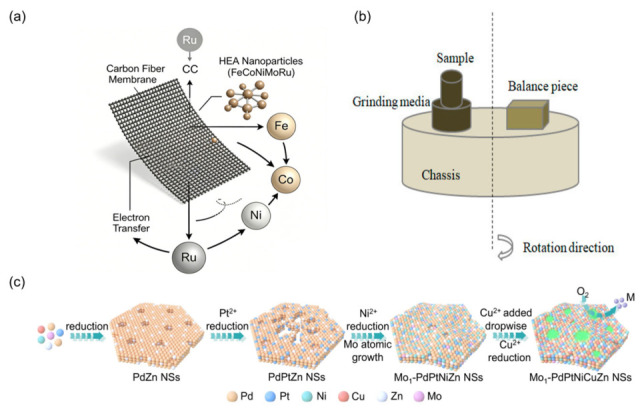
(**a**) The synthesis of FeCoNiMoRu-HEA/C on CC. (**b**) Schematic diagram of mechanical alloying method [57]. (**c**) Schematic illustration of the formation mechanism of Mo1-PdPtNiCuZn SAHEA [56].

The multi-element synergy in HEAs can optimize the adsorption energy of reactants, thereby reducing the reaction overpotential. Zhang et al. [51] fabricated a FeCoNiMoCr HEA thin film electrode on a Ti substrate. The electrode featured a rough surface with uniform elemental distribution. The rough surface increased the electrochemically active surface area, the multiple transition metals synergistically optimized the adsorption energy of intermediates, and the high-entropy and sluggish diffusion effects enhanced structural stability. Its hydrogen evolution reaction (HER) performance was significantly superior to that of the Ti substrate and noble metal RuO_2_, proving that its intrinsic catalytic activity surpasses that of a single noble metal oxide. Beyond multi-element synergy, one-dimensional, two-dimensional, or porous structures can greatly enhance electrolyte infiltration and decomposition efficiency. Liu et al. [52] developed a strategy based on 3D transition-metal-regulated high-entropy alloy mesoporous nanotubes (HEA mNTs, PtPdRuIrFeCu). On the one hand, by incorporating transition metals such as Mn, Fe, Co, and Ni, they systematically optimized the catalyst’s electronic structure, oxygen adsorption energy, and d-band center position. On the other hand, the mesoporous structure facilitated mass transport, and defect sites provided highly active sites. Among them, Fe-regulated PtPdRuIrFeCu mNTs achieved a mass activity of 1.94 A/mgPt in the oxygen reduction reaction (ORR), which is 8.43 times that of commercial Pt/C. Hu et al. [53] successfully constructed a self-supporting electrode of a low-Ru-loaded FeCoNiMoRu quinary HEA on a carbon fiber membrane (CC@FeCoNiMoRu-HEA/C), using a metal–organic framework (MOF) as a precursor, for efficient catalysis of the oxygen evolution reaction (OER) in zinc–air batteries, as shown in Figure 3a. Differences in the electronegativity of the multiple metal elements induced a rearrangement of the electronic structure, with electrons transferring from Fe, Co, Ni, and Mo to the more electronegative Ru. This precisely modulated the adsorption energy of OER intermediates. Ashwinia et al. [54] proposed a strategy of compositing HEA nanoparticles with graphene (HEA-G). Using mechanical ball milling combined with ultrasonic exfoliation, as shown in Figure 3b, they prepared NiFeCrCoCu HEA-graphene composites with different mass ratios (50:50, 70:30, 90:10). By leveraging the high conductivity and large specific surface area of graphene, along with the multi-principal-element synergy and structural stability of the HEA, they significantly enhanced the catalytic performance for the urea oxidation reaction (UOR). The composite with the 70:30 ratio exhibited the best electrochemical response, achieving a sensitivity of 37.4 μA·mM^−1^·cm^−2^ for urea oxidation at an operating potential of 0.4 V, with a response time of less than 1 s. Yang et al. [55] employed a non-equimolar high-entropy strategy to prepare two novel high-entropy perovskite proton conductor materials: BaSn_0.15_Ce_0.35_Zr_0.25_Y_0.1_In_0.1_Gd_0.05_O_3_-δ (BSCZYIT) and BaSn_0.15_Ce_0.35_Zr_0.25_Y_0.1_Yb_0.1_Gd_0.05_O_3_-δ (BSCZYYbG). These materials possess a single cubic perovskite structure and dense microstructure. At 600 °C in a water vapor and oxygen atmosphere, they exhibited a proton transport number of 0.90 and a proton mobility of 6.71 × 10^−6^ cm^2^/(V·s). In an air atmosphere, proton conduction dominated, and proton mobility significantly increased with temperature. Their high proton conductivity stems from higher proton mobility rather than proton concentration, with the high-entropy effect and multi-component synergy optimizing the lattice environment and carrier transport. This work confirms that high-entropy perovskite oxides can serve as high-performance proton conductor electrolytes. He et al. [56] reported a Mo-modified PdPtNiCuZn HEA ultrathin nanosheet (Mo_1_-PdPtNiCuZn), as shown in Figure 3c, featuring intrinsic tensile strain and isolated Pt-Pt sites. This material exhibited extremely high mass activity and excellent durability in the methanol oxidation reaction (MOR). The introduction of single-atom Mo not only optimized the electronic structure of Pt sites, inhibiting the formation of CO intermediates, but also steered the reaction pathway towards a formate route. Combined with tensile strain, this synergistically lowered the reaction energy barrier, significantly enhancing CO tolerance and catalytic efficiency.

In summary, rational composition design, morphology control, defect engineering and interface modification enable HEAs to realize synergistic optimization of electrocatalytic activity, selectivity, stability and conductivity. These characteristics perfectly match the core requirements of sensitive interfaces in electrochemical sensors. In the detection of biomolecules, gases, heavy metal ions and environmental pollutants, HEAs can function as catalytic active sites for sensing reactions, electron/ion transport channels, stable supporting skeletons and anti-interference interfacial layers. Therefore, HEAs are promising functional materials for developing next-generation electrochemical sensors with high sensitivity, good selectivity and long-term stability.

## 3. Controllable Fabrication of HEA Micro/Nano Structures

### 3.1. HEA Core Processes for HEA-Based Flexible Sensor Sensitive Layers

Thin-film micro/nano structures are the primary morphology for constructing the sensitive layers of flexible sensors. HEAs are commonly applied in the form of alloy foils [58,59,60], flexible fibrous membranes [61,62,63] and electrode coatings [64,65,66]. Achieving precise thickness control, uniform composition, and good compatibility with flexible substrates are the core objectives of the fabrication processes for these alloy thin films. The main preparation methods include mechanical alloying, wet-chemical synthesis, and non-equilibrium synthesis.

#### 3.1.1. Mechanical Alloying

Mechanical alloying achieves alloying of different metal or alloy powders in the solid state through physical means such as high-energy ball milling or mechanical attrition. The core mechanism involves using the localized high temperatures and pressures from high-speed collisions to drive interdiffusion of atoms, thereby forming supersaturated solid solutions [67,68,69]. This method can induce a large number of dislocations and grain boundaries in HEAs, significantly enhancing their mechanical properties. Simultaneously, by precisely controlling the milling time, nanocrystalline structures with grain sizes below 100 nm can be obtained, which shortens charge carrier transport paths and thus improves the electrical response speed of the sensor. Rajendrachari’s team [64,65] has employed mechanical alloying (high-energy ball milling) to prepare HEA powders, using them as sensitive modifiers for carbon paste electrodes in electrochemical sensing. For example, 23Fe-21Cr-18Ni-20Ti-18Mn HEA was used for ascorbic acid (AA) detection, while 20Fe-20Cr-20Ni-20Mn-20Ti HEA was used for methyl orange (MO) detection. Their studies show that nanocrystalline HEA powders prepared by mechanical alloying significantly enhance the electrochemical sensing performance towards target analytes by increasing the electrode’s active area and leveraging the synergistic catalytic effects among the multiple transition metals.

#### 3.1.2. Wet-Chemical Synthesis

Wet-chemical methods deposit thin films via chemical reactions in solution, offering advantages such as low cost, simple equipment, and ease of large-area preparation. Ni et al. [61] successfully prepared ultrathin PtRuFeCoRhW senary HEA nanorods (HEANRs) using a solvothermal co-reduction strategy. They constructed a colorimetric sensing platform for the detection of 2-mercaptobenzothiazole (2-MBT) based on the peroxidase-like activity of these nanorods. This method achieved atomic-level homogeneous mixing of six metals and controlled growth of a 1D rod-like morphology through the co-reduction of multi-metal precursors under solvothermal conditions, combined with the spatial confinement effect of surfactants. This work represents a typical example of using wet-chemically synthesized multi-element HEA nanozymes for small-molecule pollutant detection. Its core advantages lie in the solvothermal co-reduction enabling atomic-level homogeneous mixing and morphological control. Lv et al. [62] developed a one-pot wet-chemical co-reduction method. Using H_2_ bubbles, generated in situ during the rapid reduction of metal precursors by NaBH_4_, as dynamic templates, they successfully synthesized a self-supporting PtPdMnCoFe quinary HEA nanochain network (HEAINN). This was used to construct an ultrasensitive electrochemical immunosensor for label-free detection of the biomarker neuron-specific enolase (NSE).

#### 3.1.3. Non-Equilibrium Synthesis

Non-equilibrium methods involve rapid solidification or deposition under non-equilibrium conditions, allowing materials to retain crystal structures or high-energy phases that are difficult to form under conventional conditions. The primary techniques are magnetron sputtering and laser deposition. Tan et al. [63] fabricated a near-equiatomic NiFeCrCo HEA target by laser cladding and deposited thin films (~90–850 nm) on Si, sapphire, PI, and PET substrates via magnetron sputtering. The films exhibited a single FCC structure with uniform composition (Ni 24.0%, Fe 25.4%, Cr 25.9%, Co 24.7%) and no secondary phases. Compared to spark plasma sintering, laser cladding offers shorter processing time, higher target density, and better compositional homogeneity. Uporov et al. fabricated a series of refractory BCC high-entropy alloys (TiZrHfNb [58], TiZrHfNbTa [59], and TiZrHfTa [60]) by arc-melting pure elemental metals (≥99.99 wt%) under a flowing helium atmosphere, followed by at least ten remelting cycles to ensure chemical homogeneity. For strain gauge testing, thin foils (~200 μm thick) were prepared by cold rolling with approximately 98% thickness reduction. The as-cast alloys exhibited a single-phase BCC structure with dendritic microstructures and uniform elemental distribution. Electrical resistivity measurements under hydrostatic pressure (up to ~5.5 GPa) and uniaxial tension revealed that these alloys possess high resistivity with weak temperature dependence and superior gauge factors (GF = 3.49–5.17), outperforming commercial Constantan and Manganin. Ab initio calculations indicated that elastic anisotropy, rather than changes in electronic structure, dominates the strain sensitivity.

### 3.2. Comparison of Different Fabrication Processes and Their Suitability for Flexible Applications

Different fabrication processes have their own advantages and disadvantages in terms of material system applicability, structural control capability, cost, and scalability. The choice must be based on the specific application scenario of the target flexible device. Magnetron sputtering excels in film uniformity, composition control precision, and compatibility with photolithography [70,71]. It is suitable for producing sensitive layers for high-precision, high-consistency strain and temperature sensors. However, it involves high equipment investment and relatively low material utilization (especially with planar targets) and faces challenges in achieving uniform deposition on curved or irregular flexible substrates. Laser melting/additive manufacturing has unique advantages for rapid prototyping and complex 3D structure formation, but its direct application on flexible substrates is currently limited by high heat input, making it more suitable for mold preparation or as an intermediate step in transfer printing processes [72,73].

For 1D structure fabrication, cold drawing combined with current-assisted processing is suitable for producing continuous, high-strength, and tough micron-scale fibers, making it particularly attractive for fiber-shaped devices in smart textiles and wearable electronics. However, this approach requires alloys with excellent workability and is limited in its ability to reduce fiber diameters to the submicron scale. Electrospinning, on the other hand, enables the fabrication of ultra-fine nanoscale fibers and is especially advantageous for constructing high-entropy alloy/carbon-composite-sensitive layers with large specific surface areas [74,75]. This technique holds great promise for flexible gas sensors and electrochemical biosensors, and offers potential for large-scale production. Nevertheless, the random orientation and non-woven morphology of electrospun fibers restrict their application in devices requiring precise alignment or highly ordered arrays.

For the fabrication of nanopowders/functional structures, mechanical alloying is a simple, low-cost, and scalable method, representing the most economical approach for producing high-entropy alloy powders. However, the resulting powders typically exhibit irregular morphology and a broad size distribution, often necessitating post-treatment to meet the stringent requirements of high-performance sensors. While mechanical alloying shows significant potential in gas sensing applications [76,77,78], its current limitations include low yield and wide particle size distribution. In contrast, wet-chemical synthesis offers distinct advantages in the precise control of nanoparticle size, morphology, and surface state, making it well-suited for the preparation of high-performance sensing materials. Nonetheless, its scalability is constrained by reactor volume, the need for surfactant removal, and post-synthesis purification steps, resulting in relatively higher costs.

Overall, the fabrication processes for HEA micro/nano structures for flexible sensing applications are evolving from single methods towards the integration of multiple technologies. Future trends include combining wet-chemical synthesis with printing techniques for direct patterning of flexible sensors; integrating electrospinning with carbonization to build self-supporting flexible electrode materials; and combining magnetron sputtering with elastic substrates to develop highly stretchable island-bridge structured sensing arrays. With continuous optimization of process parameters and a deepening understanding of the composition–structure–process–performance relationships in HEAs, their application in flexible sensing is gradually moving from laboratory research to industrial practice (Table 1).

## 4. Applications of HEA-Based Flexible Sensors

### 4.1. HEA-Based Flexible Strain/Stress Sensors

Materials for strain sensors must comprehensively meet multiple mechanical and electrical requirements, including high strength and good plasticity, high resistivity and high gauge factor, and a stable temperature coefficient of resistance. Based on morphological differences, strain sensors can be categorized into resistive and capacitive types, with resistive sensors being widely used due to their simple structure and strong anti-interference capability. Numerous studies have confirmed that HEAs can simultaneously possess multi-faceted properties. The presence of numerous nanotwin boundaries and grain boundaries within HEAs contributes to their high hardness and tunable elastic properties. Nanotwins have been widely demonstrated to provide significant strengthening in metals and alloys, while the high density of grain boundaries and severe lattice distortion further increase the electrical resistivity of the alloy. Consequently, HEAs are ideal materials for resistive sensors [79,80].

In terms of material preparation and property control, Feng et al. [81] explored a novel WNbMoTaV high-entropy thin film (HETF) with an amorphous structure using a pulsed laser deposition system. The film formed a BCC crystalline structure, achieving a synergy of high resistivity and excellent corrosion resistance. The high resistivity (~320.6 μΩ·cm) is primarily attributed to s-d scattering effects of transition metals, chemical disorder, and electron scattering caused by the amorphous structure. The excellent corrosion resistance (low corrosion current density of 0.088 μA/cm^2^) originates from the cocktail effect, the absence of grain boundaries in the amorphous structure, and the protective effect of stable oxide films (from Nb, Mo, Ta) and bound water. This film shows great application potential in microelectronic devices as resistive sensors. Hsu et al. [82] fabricated high-entropy NbMoTaW alloy thin films on silicon substrates via radio frequency (RF) magnetron sputtering, systematically analyzing the physical and conductive properties under different substrate temperatures and RF powers. As the substrate temperature increased to 300 °C, the grain size increased, and the surface morphology evolved from dense and smooth to a leaf-like structure, attributed to enhanced surface diffusion of adatoms and grain nucleation/growth at higher temperatures. In terms of electrical properties, the optimized film achieved a low resistivity of 58 μΩ·cm, significantly better than films of the same composition prepared by DC sputtering (~170 μΩ·cm). This improvement is due to reduced grain boundary scattering and improved crystalline quality from grain growth at higher substrate temperatures. This material system combines the high-temperature stability of refractory metals with tunable electrical conductivity, making it suitable as an electrode material for sensors.

**Figure 4:**
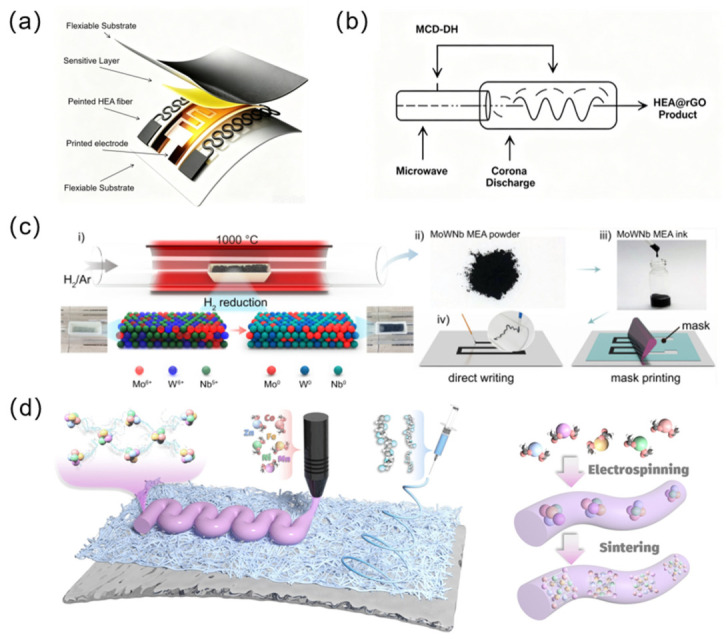
(**a**) Schematic diagram of a flexible strain sensor based on HEA. (**b**) Schematic diagram of microwave corona-discharge-assisted microwave heating (MCD-MH). (**c**) Fabrication of flexible painting electronic devices and integrated systems with MoWNb MEAs inks [85]. (**d**) Schematic diagram of the sensor preparation process [86].

In terms of composite system design, Zhang et al. [83] proposed a high-entropy alloy-reinforced composite strain sensor (PHM-FPSS), synergistically incorporating CoCrFeMnNi HEA powder and multi-walled carbon nanotubes (MWCNTs) into a PDMS elastomer matrix. The design uses HEA particles as mechanically stable conductive bridging nodes and MWCNTs to build a continuous 3D conductive network, forming a “flexible conductive network + rigid load-bearing node” synergistic structure. The sensor achieved a response time of 88 ms and a recovery time of 115 ms. Leveraging its excellent fatigue resistance and mechanical stability, it overcomes the bottleneck of poor long-term cycling stability in traditional carbon/metal-based sensors. Wan et al. [84] proposed a rapid synthesis strategy involving microwave corona-discharge-assisted microwave heating (MCD-MH), as shown in Figure 4b. This approach achieved precursor decomposition, graphene oxide reduction, and the in situ construction of an AlCoCrFeNi HEA nanoparticle-reduced graphene oxide core–shell structure in seconds under ambient air. This core–shell structure, through an elastic capillary engulfing effect induced by flash photon sintering, embedded the HEA particles into the PDMS substrate, forming a mechanically interlocked conductive network. This resulted in a wide strain range (0.1–30%), a high gauge factor (up to 295.9), fast response (<100 ms), and excellent cycling stability (over 10,000 cycles).

In terms of direct printing and flexible integration, Li et al. [85] reported a direct ink writing (DIW) strategy based on a MoWNb medium-entropy alloy (MEA) ink. This method allows the direct fabrication of flexible strain sensors on arbitrary complex surfaces without complex post-treatment, as shown in Figure 4c. The sensor features an ultra-wide operating temperature range (−150 °C to 1100 °C), a high sensitivity coefficient of −752.7 at 300 °C, a low detection limit of 0.57 microstrain, and excellent thermal stability and long-term reliability. Zheng’s team [86] successfully fabricated FeCoNiMnZn HEA nanofibers within a PVDF nanofiber membrane using electrohydrodynamic direct writing and subsequent annealing metallization, forming a uniform conductive network. This flexible strain sensor exhibited an extremely low temperature coefficient of resistance (45.59 ppm/K) in the −10 °C to 70 °C range, a gauge factor of 1.12 (within 50% strain), a response time of 310 ms, and maintained stability over 6000 stretching cycles and >1200 h of long-term testing without baseline drift. This temperature-immune characteristic originates from the inherent lattice distortion and phonon softening effects in the HEA, which effectively suppress electron–phonon scattering. The sensor was successfully applied to monitor human multi-joint motion and robotic hand grasping.

### 4.2. HEA-Based Gas Sensors

Gas sensing technology is a core enabler for environmental monitoring, industrial safety, medical diagnostics, and other fields. Its performance directly determines detection accuracy, response efficiency, and application scenario suitability. Therefore, developing gas sensors that combine high sensitivity, high selectivity, long-term stability, and a wide detection range is a pressing need and research hotspot in materials science and sensing technology. As a novel multi-component alloy composed of five or more elements in near-equimolar ratios, HEAs, with their unique high-configurational entropy, lattice distortion, and multi-element synergy, break the performance limitations of traditional single- or few-component alloys. They show irreplaceable potential in catalysis and sensing [87,88,89], offering a new materials solution for breakthroughs in gas sensor performance and have recently become a research frontier in the field.

**Figure 5:**
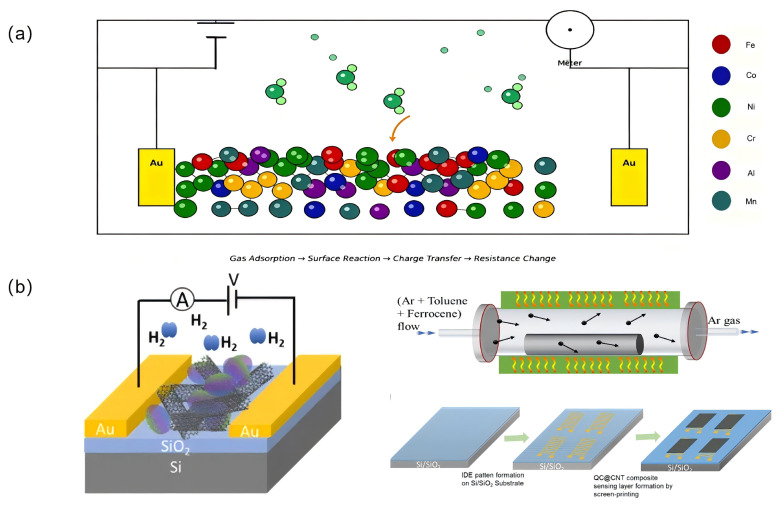
(**a**) Schematic diagram of a flexible gas sensor based on a high-entropy alloy. (**b**) Schematic of QC@CNT sensor fabrication by the screen-printing technique on SiO_2_/Si substrates using Au/Cr IDE patterns [90].

For hydrogen (H_2_) sensing, palladium-based HEAs are a primary choice. Kumar et al. [90] developed a room-temperature hydrogen sensor based on an AlMnPdPtAu HEA quasicrystal nanosheet and carbon nanotube composite, fabricated using a low-cost screen-printing process, as shown in Figure 5b. The sensor achieved relative responses of 103% and 130.4% to 1 ppm and 100 ppm H_2_ at room temperature, respectively, with a response time of only 19 s and a detection limit as low as 31 ppb. It also exhibited excellent selectivity and long-term stability. Structural and characterization analyses confirmed the formation of an effective heterointerface between the quasicrystal and CNTs. Density functional theory (DFT) calculations revealed the dissociative adsorption mode and spillover effect of hydrogen at the quasicrystal/CNT (QC@CN) interface, where hydrogen atoms bond with Mn atoms in the quasicrystal, facilitating hydrogen migration and stabilization. This provides a new material design approach for high-performance room-temperature hydrogen sensing. Du et al. [91] successfully synthesized a composite structure of PdPtMnCoNi HEA nanoparticles (3–10 nm) decorated on porous Nb_2_O_5_ microspheres via hydrothermal and liquid-phase reduction methods. The HEA/Nb_2_O_5_ sensor exhibited excellent H_2_ sensing performance at 175 °C: a response of 28.5% to 400 ppm H_2_ (35 times higher than pure Nb_2_O_5_ at 0.8%), a response time of only 3 s (for 1000 ppm H_2_), a low detection limit of 5 ppm, a wide detection range of 5–10,000 ppm, and outstanding selectivity. The performance enhancement mechanism is attributed to the strong catalytic activation of H_2_ and O_2_ by the HEA and the Schottky barrier formed at the HEA/Nb_2_O_5_ interface, which effectively modulates charge transport and depletion layer thickness. This demonstrates that HEA decoration is an effective strategy to boost the performance of oxide semiconductor gas sensors.

For nitrogen oxides (NO_2_), Mishra et al. [92] successfully prepared 2D nanosheets (~6 nm thick) of a decagonal quasicrystal with composition Al_70_Co_10_Fe_5_Ni_10_Cu_5_ via liquid-phase exfoliation and applied them for NO_2_ gas sensing for the first time. The 2D quasicrystal sensor showed a response of 46% to 100 ppm NO_2_ at 100 °C, a detection limit as low as 1 ppm, and excellent selectivity towards NO_2_ compared to interfering gases like NH_3_, H_2_S, H_2_, and SO_2_. The sensing mechanism is based on NO_2_, as an electron acceptor, extracting electrons from the 2D quasicrystal, increasing hole concentration, and decreasing resistance. DFT calculations further showed that NO_2_ has a high adsorption energy (−289 to −233 meV/atom) on the quasicrystal surface, while H_2_ adsorbs much more weakly, explaining the material’s excellent anti-interference properties.

Combining HEAs with 2D materials is another effective strategy to enhance gas sensing performance. Kusuma Urs MB’s team [93] prepared Ag-Au-Cu-Pd-Pt HEA nanoparticles via a low-temperature grinding process and achieved uniform decoration of MoS_2_ with HEA using ultrasonic dispersion, constructing a MoS_2_-HEA composite system for high-performance hydrogen sensors. Oxygen species (O^2−^) adsorbed on the MoS_2_ surface extract electrons, giving the composite p-type conductive characteristics. When reducing H_2_ gas adsorbs, it recombines with holes in the system, causing a significant increase in resistance, enabling precise H_2_ detection. In this system, elements like Pd and Pt in the HEA have strong dissociative adsorption capacity for H_2_, while Ag and Au promote the spillover of H atoms to the MoS_2_ surface, increasing active sites and adsorption efficiency. Simultaneously, the Schottky junction formed between the HEA and MoS_2_ causes small changes in carrier concentration to induce significant resistance fluctuations, amplifying the sensing signal and improving sensitivity. Bidesh Mondal et al. [94] also used non-noble metal Ti-Zr-V-Nb-Hf HEA nanoparticles to decorate MoS_2_, increasing the sensor’s sensitivity from 7% to 47%. This strategy leverages the high catalytic activity from multi-element synergy in the HEA and the large specific surface area of MoS_2_, constructing an efficient “adsorption-dissociation-charge transfer” sensing chain and providing new ideas for developing low-cost, low-power, high-performance gas sensors.

### 4.3. HEA-Based Electrochemical Sensors

In the field of electrochemical sensing, HEAs show great promise due to their unique compositional tunability and excellent electrocatalytic performance [95,96,97,98]. Their catalytic mechanism originates from the unique “active site map” constructed by multi-principal elements on the surface: atoms of different elements synergistically optimize the adsorption energy of the target molecule and the desorption energy of intermediates, thereby lowering the reaction energy barrier. Simultaneously, severe lattice distortion leads to highly heterogeneous coordination environments for surface atoms, inducing a large number of highly active step and defect sites. The synergistic action of these structural features significantly accelerates the electrooxidation or electroreduction kinetics of typical sensing targets like glucose, hydrogen peroxide, and heavy metal ions.

In biomedical detection, Feng’s team [99] designed a split-type photoelectrochemical (PEC) aptasensor for the ultrasensitive detection of the cardiac biomarker myoglobin. They used ultrathin PtCoFeRuMo HEA nanowires as a peroxidase-mimicking nanozyme to catalyze the precipitation of 3-amino-9-ethylcarbazole (AEC) for signal amplification, combining this with a Z-type WO_3_/ZnIn_2_S_4_ heterojunction photoelectrode to significantly enhance photoelectric conversion efficiency and electron separation. The sensor showed a good linear range from 1.0 × 10^−2^ to 1.0 × 10^5^ pg mL^−1^ and a low detection limit of 0.96 fg mL^−1^, along with excellent selectivity, stability, and applicability in real serum samples. This provides a new approach for using HEA nanozymes in PEC bioanalysis. Building on this, the same team constructed a split-type PEC aptasensor [100] based on hollow In_2_S_3_/WO_3_ heterostructures and PtRuFeCoW HEA nanorods for the ultrasensitive detection of the highly toxic mycotoxin patulin. The sensor exhibited a good linear relationship from 0.1 pg mL^−1^ to 500 ng mL^−1^ and a low detection limit of 0.063 pg mL^−1^ and was successfully applied for spike-and-recovery detection of patulin in apple juice samples. Wang et al. [101] designed a chemical DNA biosensor based on a lightweight AlCrMnZnAu_0.1_ HEA. Using a geometric and catalytic dual-signal-amplification strategy, they achieved ultrasensitive simultaneous detection of the human papillomavirus (HPV) genotypes HPV-16/18 in human serum. The HEA was synthesized via coordination-driven synthesis using folic acid and histidine-functionalized boron–nitrogen-doped graphene quantum dots as a template, exhibiting catalytic activity 6.8 times higher than conventional gold nanoparticles. Combining target DNA recycling and thionine/ferrocene redox signal enhancement, the sensor showed a linear detection range of 10^−18^ to 10^−13^ M for both genotypes, with a detection limit as low as 2.9 × 10^−19^ M. It also demonstrated excellent single-base mismatch discrimination and clinical sample applicability, providing a high-precision, low-cost new tool for early cervical cancer screening. Furthermore, the same team designed an electrochemical biosensor [102] based on Bi_0.1_MnFeCoNi HEA nanoparticles for the ultrasensitive detection of the endometrial-cancer-related biomarker microRNA-200a-3p. The HEA, with an FCC/B2 dual-phase structure, was synthesized via coordination-driven synthesis and two-step thermal annealing and uniformly dispersed in a 3D graphene conductive scaffold. Due to multi-metal synergy and Bi-induced lattice strain, the material showed electrocatalytic activity over five times higher than that of gold nanoparticles.

**Figure 6:**
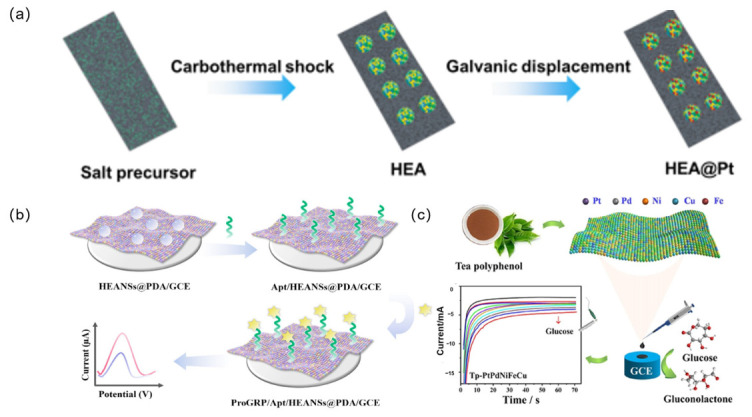
(**a**) Schematic for the preparation process of HEA@Pt [103]. (**b**) Schematic representation of the ProGRP aptasensor [104]. (**c**) Schematic for PtPdNiFeCu High-Entropy Alloy Nanoparticles for Glucose Detection [105]. The multicolored curves represent responses under different glucose concentrations to visually reflect the variation trend of sensing signals.

Liang et al. [103] developed a multifunctional electrochemical sensing platform based on FeCoNiCu high-entropy alloy-supported platinum clusters (HEA@Pt) and combined it with machine learning algorithms to achieve high-precision simultaneous detection of mixtures of dopamine, uric acid, and acetaminophen. In this material, Pt is uniformly anchored as ultra-small clusters on the HEA surface, showing excellent electrocatalytic activity towards all three analytes. The detection limits were as low as 0.037, 0.74, and 0.058 μM, respectively, with good recovery in serum samples, as shown in Figure 6a. Chandio et al. [104] developed an electrochemical aptasensor based on two-dimensional high-entropy alloy nanosheets (HEANSs) functionalized with polydopamine (PDA) for the ultrasensitive detection of ProGRP, a key biomarker for small cell lung cancer, as shown in Figure 6b. Ultrathin HEANSs composed of Fe, Co, Cu, Ni, and Ir were synthesized via a salt-template method. Their multi-metal synergy and lattice distortion provide high conductivity, abundant active sites, and excellent stability. The PDA coating introduces abundant functional groups, enhancing aptamer immobilization efficiency and biocompatibility. The constructed sensor showed a linear detection range for ProGRP from 10 pg mL^−1^ to 100 μg mL^−1^, a low detection limit of 0.874 pg mL^−1^, and a relative standard deviation of 1.78% and retained 96.25% of its initial signal after 9 days of storage. Recovery rates in human serum samples ranged from 96.8% to 98.0%. Wang et al. [105] developed a one-step hydrothermal method using tea polyphenols as both a reducing agent and a stabilizer to synthesize PtPdNiFeCu HEA nanoparticles (Tp-PtPdNiFeCu HEA-NPs) for non-enzymatic glucose detection. The as-prepared nanoparticles exhibit an average diameter of approximately 7.5 nm, a uniform face-centered cubic (fcc) structure, and homogeneous elemental distribution, as shown in Figure 6c. Electrochemical evaluations demonstrate that the sensor achieves a wide linear range from 0 to 10 mM, a sensitivity of 1.264 μA·mM^−1^·cm^−2^, and a low detection limit of 4.503 μM. Furthermore, the sensor shows excellent anti-interference capability against common coexisting species such as ascorbic acid, uric acid, acetaminophen, and NaCl, along with remarkable stability over 50 consecutive cyclic voltammetry scans. This work validates the feasibility of green synthesis routes for producing high-performance HEA-based electrochemical sensors and opens new avenues for portable and biocompatible glucose sensing devices.

Regarding enzyme-like catalytic mechanisms, Yang et al. [106] reported a RuPtIrRhCu quinary HEA nanozyme (HEAzyme) designed for significantly enhanced peroxidase (POD)-like activity. Their study found that the strong adsorption capacity of Ru sites for H_2_O_2_ and hydroxyl radicals (OH), combined with the multi-element synergistic effect, enabled rapid transfer of OH from Ru to neighboring sites like Pt. This prevents blockage of active sites and significantly lowers the catalytic reaction energy barrier. The HEAzyme maintained its activity completely after 6 months of storage at room temperature, demonstrating superior catalytic activity and stability compared to single-metal nanoparticles and natural horseradish peroxidase. A multi-channel colorimetric sensor array based on this HEAzyme successfully distinguished eight different bio-antioxidants.

For hydrogen peroxide detection, Qin’s team [107] fabricated a hierarchically nanoporous CoCrFeNiAl_1.5_ high-entropy alloy electrode (NPCCF-1.5) via selective phase dealloying. The material features a bimodal pore structure with large pores (~580 nm) and small pores (~13 nm), along with in situ retained Fe-Cr(Co)-rich grain boundaries. This structure provides abundant catalytic active sites (Co, Cr, Fe oxides) and efficient mass transport channels, while the grain boundaries accelerate the diffusion of electrons and reactant molecules. The sensor achieved a high sensitivity of 510.4 μA mM^−1^ cm^−2^ for H_2_O_2_, a wide linear range up to 49.95 mM, and a low limit of quantification of 4 μM (S/N = 3), along with good anti-interference ability, long-term stability, and reproducibility. 

In food safety detection, Qian et al. [108] employed an “HEA-carbon-based support” composite strategy, anchoring FeCoNiCrCe HEA nanoparticles in situ onto carbon nanofibers. The confinement effect of the carbon nanofibers inhibited nanoparticle agglomeration, and the synergy between the two components enhanced conductivity and active site density. This enabled the simultaneous sensitive detection of three heavy metal ions (Cd^2+^, Pb^2+^, Cu^2+^) in fruits and vegetables. Feng’s team [109] developed PtPbBiFeMn HEA nanosheets with a well-defined morphology via a one-pot wet-chemical co-reduction method. Utilizing their excellent peroxidase-like activity, they constructed a dual-mode sensor for organophosphorus pesticide (OP) detection. The HEA nanosheet nanozyme catalyzed the oxidation of tetramethylbenzidine (TMB) and o-phenylenediamine (OPD), achieving a detection limit of 6.63 × 10^−4^ mg kg^−1^ for OPs. The sensor showed good recovery and anti-interference ability in juice and lettuce samples, and the “cocktail effect” from multi-metal synergy provides a new strategy for the rapid detection of OPs in food and environmental samples.

## 5. Discussion and Outlook

### 5.1. Comparison of HEAs with Conventional Alloys

Conventional metallic electrodes are mostly optimized to pursue ultralow resistivity, yet their electrical transport behaviors (including the temperature coefficient of resistance, TCR) are dominated by one single matrix element [39,86], resulting in narrow modulation windows. For example, pure metals generally feature large positive TCR values, which severely limit their deployment in wide-temperature-range sensing systems. By contrast, the electronic band structure of HEAs is co-regulated by multiple elemental components, enabling flexible customization of resistivity, TCR, and piezoresistive sensitivity. Rational compositional adjustment allows HEAs to act either as stable electrode layers with suppressed TCR drift or high-performance piezoresistive sensing media, accomplishing a functional transition from simple passive conductive layers to stimuli-responsive active sensing components.

For biomedical wearable and industrial monitoring applications, sensing devices frequently endure harsh service conditions including sweat-induced electrochemical erosion, high-temperature oxidation, and acidic/alkaline chemical corrosion [110,111,112]. Copper-based conductors readily oxidize under ambient conditions, whereas silver thin films suffer from electromigration and sulfidation degradation. Benefiting from the sluggish diffusion and cocktail effects intrinsic to multi-element solid-solution HEAs, compact protective passivation layers spontaneously form on HEA surfaces, endowing them with outstanding antioxidation and anticorrosion capacity. Moreover, unlike liquid metals that carry inherent leakage hazards, solid-state HEAs avoid fluid outflow risks and demonstrate greater reliability for long-term wearable and implantable biomedical sensors. A quantitative performance comparison of state-of-the-art alloy-type flexible sensing materials, corresponding application scenarios, and core characteristic parameters is summarized in Table 2.

### 5.2. Current Challenges for HEA-Based Sensors

Despite the remarkable laboratory-scale progress, several critical challenges hinder the practical, industrial-scale application of HEA-based flexible sensors. A primary challenge lies in manufacturing scalability and cost. While techniques like magnetron sputtering and laser cladding offer excellent control, they are often capital-intensive or difficult to scale for high-throughput production. Conversely, low-cost methods like mechanical alloying produce powders with ill-defined morphologies, requiring post-treatment. A significant research thrust must therefore focus on developing scalable, cost-effective, and environmentally benign synthesis routes, such as ink-based printing (e.g., electrospinning, inkjet printing) combined with novel HEA ink formulations and rapid post-processing [120,121,122,123].

A second challenge is long-term mechanical and electrical stability under real-world dynamic deformation [124,125,126]. Integrating HEA micro/nanostructures into flexible devices also faces notable challenges. Mismatch in mechanical properties between HEA micro/nanomaterials and flexible substrates easily causes interfacial delamination under repeated bending and stretching. In addition, the structural evolution of HEAs after deformation may alter electron transport and electrocatalytic activity, leading to drifting sensing signals. It is also difficult to achieve uniform deposition and patterning of HEA micro/nanostructures on curved or irregular flexible surfaces, which further undermines device consistency and long-term reliability.

A third challenge is that the extreme compositional complexity of HEAs also poses prominent challenges. The enormous elemental combination space leads to a huge number of potential alloy formulas, making the traditional trial-and-error method extremely inefficient for screening optimal sensing materials. Moreover, subtle variations in elemental ratios can cause dramatic changes in phase constitution, microstructure and electronic states, resulting in poor repeatability of material properties. In addition, the complex multi-element system easily induces unpredictable phase separation and elemental segregation during synthesis and post-processing, which further hinders the precise control of sensing performance and large-scale industrial production [127,128,129].

### 5.3. Future Outlook

The trajectory of HEA-based flexible sensors points toward several exciting and transformative directions. First, the integration of machine learning (ML) and high-throughput screening will revolutionize the discovery and optimization of HEAs. Combined high-throughput experimental screening and ML form an efficient data-driven design pipeline for flexible HEA sensors. Different from traditional empirical trial-and-error, this integrated workflow rapidly filters candidate HEA compositions; predicts strain sensitivity, fatigue durability and electrical stability; and optimizes micro-nano fabrication parameters, greatly shortening the cycle of developing high-performance flexible sensing materials [130,131,132].

Second, the development of multi-functional and self-powered sensing systems represents a key application frontier. Future devices will likely integrate sensing, data storage, and energy harvesting capabilities into a single, flexible platform. HEAs, with their tunable electrochemical and mechanical properties, are ideal candidates for such integrated systems, potentially functioning as both the sensing element and the electrode in a flexible energy harvester.

Third, for practical biomedical and robotic applications, biocompatibility and conformability will become paramount. Research is needed to develop HEAs based on non-toxic, bio-friendly elements and to engineer their surfaces or couple them with soft, bioresorbable substrates. The ultimate goal is to create sensors that not only match the mechanical properties of biological tissue (low modulus, high stretchability) but also exhibit seamless, long-term stability.

In conclusion, while significant challenges remain in manufacturing, stability, and design complexity, high-entropy alloys have unequivocally established themselves as a transformative material platform for next-generation flexible sensors. By strategically addressing these challenges through interdisciplinary collaboration—combining materials science, data-driven discovery, and advanced manufacturing—the field is poised to deliver high-performance, durable, and intelligent sensing solutions for personalized healthcare, environmental monitoring, and human–machine interaction.

## Figures and Tables

**Table 1:** Comparison of typical fabrication processes for HEA micro/nano structures for flexible sensors.

Alloy Composition	Key Advantages	Main Limitations	Ref.
Magnetron Sputtering	Excellent film uniformity; precise composition control; compatible with photolithography; high consistency	High equipment investment; low material utilization (planar targets); challenges with curved/irregular flexible substrates	[63,70,71]
Laser Melting/Additive Manufacturing	Rapid prototyping; capable of forming complex 3D structures; unique for mold preparation	High heat input limits direct application on flexible substrates; currently more suitable as an intermediate step	[31,72,73]
Electrospinning	Fabricates ultra-fine nanoscale fibers; large specific surface area; advantageous for HEA/carbon composites; potential for large-scale production	Random orientation and non-woven morphology restrict use in devices requiring precise alignment or ordered arrays	[74,75]
Mechanical Alloying	Simple, low-cost, and scalable; most economical approach for producing HEA powders	Irregular powder morphology and broad size distribution; low yield; often requires post-treatment	[64,65]
Wet-Chemical Synthesis	Precise control over nanoparticle size, morphology, and surface state; well-suited for high-performance sensing materials	Scalability constrained by reactor volume; requires surfactant removal and post-synthesis purification; relatively higher cost	[61,62]

**Table 2:** Comparison of alloy-based sensor materials, applications, and key performance.

Alloy Composition	Application	Key Performance	Ref.
CoCrFeMnNi powder	Flexible strain sensor	Resistivity: 0.851 Ω·m; Tensile strength: 1.97 MPa; Elongation: 279.86%; GF: 0.917 (0–15% strain), 0.280 (15–70% strain); Response: 88 ms; Recovery: 115 ms; Stable over 11,000 cycles	[83]
FeCoNiMnZn fiber	Flexible strain sensor	TCR = 45.59 ppm/K; GF = 1.12 at 50% strain; Response: 310 ms; No drift over 6000 cycles; stable over wide temperature range	[86]
Fe NWs	Flexible strain sensor	GF = 37–53 (15–57.5% strain); Nonlinearity error: 2.45%; Hysteresis: 8.3%	[113]
Ga-In alloy	Flexible strain sensor	Stretchable to 100% strain; GF increases from 1.96 to 4.36 with strain; Young’s modulus: 43.3 kPa	[114]
AlMnPdPtAu HEA/CNT	H_2_sensor	Response: 103% (1 ppm H_2_) to 130.4% (100 ppm H_2_) at RT; Response time: 19 s; Recovery: 81 s; LOD: 31 ppb; Good selectivity; stable over 4 weeks at 90% RH	[90]
Ag-Au-Cu-Pd-Pt/MoS_2_	H_2_sensor	Response: ~40% (ΔR/R) to 5000 ppm H_2_ at 80 °C; Response: 600 s; Recovery: 420 s; Stable over 0–60% RH, 1 week	[93]
Pd-Ni alloy film	H_2_sensor	Response time: 11 s to 1% H_2_ at RT; Stable over 90 days; Reversible over 5 cycles	[115]
PdAu alloy nanowire	H_2_sensor	Detection range: 0–20% H_2_; Can resolve <0.5% H_2_; Saturation above 10% H_2_	[116]
FeCoNiMnCr HENA	Electrochemical (Glucose)	Sensitivity: 3043 μA·mM^−1^·cm^−2^ (0–1 mM), LOD: 132 μM; 932 μA·mM^−1^·cm^−2^ (1–13 mM); Good anti-interference; stable for 3 h	[117]
CoCrFeNiAl_1.5_	Electrochemical (H_2_O_2_)	Sensitivity: 510.4 μA·mM^−1^·cm^−2^; Linear range: 0.05–49.95 mM; LOD: 4 μM; Response <5 s; Signal decay only 0.7% over 4000 s; RSD = 3.07%	[107]
NiPt alloy NPs	Electrochemical (Glucose)	Sensitivity: 1.824 μA·μM^−1^·cm^−2^ (0.5 μM-2.1 mM); 0.467 μA·μM^−1^·cm^−2^ (2.1–5.6 mM); LOD: 0.03 μM	[118]
AuPt alloy NPs/MoS_2_	Electrochemical (H_2_O_2_)	Sensitivity: 0.1105 mA·cm^−2^·mM^−1^ (0.05–1.05 mM); 0.0690 (1.05–3.15 mM); LOD: 0.01 mM; Retains 84.6% after 50 bending cycles, 83.2% after 15 days; RSD = 8.5%	[119]

## Data Availability

No new data were created or analyzed in this study. Data sharing is not applicable to this article.

## Abbreviations

The following abbreviations are used in this manuscript:

HEAs	High-Entropy Alloys
TCR	Temperature Coefficient of Resistance
HER	Hydrogen Evolution Reaction
ORR	Oxygen Reduction Reaction
OER	Oxygen Evolution Reaction
UOR	Urea Oxidation Reaction
PEC	Photoelectrochemical
MOF	Metal–Organic Framework
DFT	Density Functional Theory
MD	Molecular Dynamics
ECT	Electric Current Treatment
FCC	Face-Centered Cubic
BCC	Body-Centered Cubic
GF	Gauge Factor
LOD	Limit of Detection
ML	Machine Learning
MWCNTs	Multi-Walled Carbon Nanotubes
PDMS	Polydimethylsiloxane
PI	Polyimide
PET	Polyethylene Terephthalate
